# The Genetic Architecture of *FUT2–FUT3* Variation and Variant Combinations Underlies the Relatively High Prevalence of the Le(a+b+) Phenotype in Thais

**DOI:** 10.1155/humu/3749006

**Published:** 2026-11-10

**Authors:** Piyathida Khumsuk, Oytip Nathalang, Wiradee Sasikarn, Kwanlawan Thongborisut, Kamphon Intharanut

**Affiliations:** ^1^ Blood Bank Thammasat University Hospital Pathumtani Thailand tu.ac.th; ^2^ Graduate Program in Biomedical Sciences Faculty of Allied Health Sciences Thammasat University Pathumtani Thailand tu.ac.th

**Keywords:** *FUT2*, *FUT3*, Lewis blood group, Le(a+b+), Thai blood donors, weakened allele

## Abstract

**Background:**

The Lewis (Le) blood group phenotype reflects the combined effects of *FUT2* and *FUT3* genetic variation. Weak‐secretor alleles associated with reduced *α*‐(1,2)‐fucosyltransferase 2 activity may permit concurrent Le^a^ and Le^b^ expression, giving rise to the Le(a+b+) phenotype. Although Le(a+b+) is relatively frequent in Thai and other East and Southeast Asian populations, its genetic architecture in Thais remains incompletely understood.

**Objective:**

This study is aimed at characterizing the *FUT2* and *FUT3* variation across Le phenotypes and investigate the genetic architecture underlying the Le(a+b+) phenotype.

**Methods:**

A total of 708 Thai blood donors were divided into two cohorts. The first included 151 donors representing all Le phenotypes for *FUT2* and *FUT3* characterization. The second included 557 donors, from whom 100 Le(a+b+) individuals were selected for *FUT2* analysis. Le phenotyping was performed by column agglutination. Genetic analysis included PCR amplification, DNA sequencing, and variant and haplotype analysis. Large *FUT2* deletions were assessed by targeted PCR and sequencing.

**Results:**

Among the 151 donors, Le(a−b+) was predominant (57.6%), followed by Le(a+b+) (23.8%), Le(a−b−) (15.9%), and Le(a+b−) (2.7%). We identified 18 *FUT2* and six *FUT3* single nucleotide variants and 26 and 10 distinct alleles, respectively. Two *FUT2* deletion alleles, *se^del^* and *se^del2^*, were identified. Le(a+b+) was associated with diverse *FUT2–FUT3* configurations, particularly the weak‐secretor *Se^390,418^* allele with functional *FUT3* backgrounds. All 100 Le(a+b+) donors carried *Se^390,418^*, either homozygously (73/100) or with nonfunctional, deleted, or functionally uncertain *FUT2* alleles. Additionally, a novel *FUT2* missense variant, c.676G>A (p.(Ala226Thr)), was identified in one donor.

**Conclusion:**

The relatively frequent Le(a+b+) phenotype in Thais reflects diverse *FUT2* variant combinations centered on the weakened *Se^390,418^* allele. The identification of *se^del^*, *se^del2^*, and a novel *FUT2* variant with uncertain functional significance further expands the known genetic diversity underlying Le phenotypes in the Thai population.

## 1. Introduction

The Lewis (Le) blood group system (ISBT 007), as defined by the International Society of Blood Transfusion (ISBT) Working Party on Red Cell Immunogenetics and Blood Group Terminology, is distinct from most blood group systems in that its glycan antigens are not synthesized by red blood cells (RBCs). Instead, they are produced by epithelial tissues, predominantly in the gastrointestinal tract [1], and released into body fluids or the interstitial space before entering the circulation. These antigens are subsequently adsorbed onto the erythrocyte membrane from the plasma [2]. The Le system currently consists of six antigens, five of which are polymorphic (Le^a^, Le^b^, Le^bH^, ALe^b^, and BLe^b^), and one of high prevalence (Le^ab^) [3].

Le antigen biosynthesis is determined by the coordinated activity of galactoside *α*‐(1,2)‐fucosyltransferase 2 [*α*(1,2)‐FucT2] and galactoside *α*‐(1,3/1,4)‐fucosyltransferase 3 [*α*(1,3/1,4)‐FucT3], which encode fucosyltransferases involved in glycan synthesis. *α*(1,3/1,4)‐FucT3, encoded by the *FUT3* (*Le*) gene, is essential for Le antigen biosynthesis, whereas the final antigenic structure is determined by the presence or absence of functional *α*(1,2)‐FucT2, encoded by the *FUT2* (*Se*) gene [4]. In individuals lacking functional *α*(1,2)‐FucT2, *α*(1,3/1,4)‐FucT3 alone modifies the precursor chain to produce the Le^a^ antigen, resulting in the Le(a+b−) phenotype in Le‐positive nonsecretors. In contrast, when both *α*(1,2)‐FucT2 and *α*(1,3/1,4)‐FucT3 are functional, sequential fucosylation generates the Le^b^ antigen. These Le structures may subsequently be modified by ABO glycosyltransferases, producing Le^b^ in blood group O individuals and ALe^b^ or BLe^b^ structures in blood groups A and B, respectively. Accordingly, Le‐positive secretors typically exhibit the Le(a−b+) phenotype. By contrast, individuals lacking functional *α*(1,3/1,4)‐FucT3 synthesize Le antigens and therefore display the Le(a−b−) phenotype regardless of secretor status [4]. At the genetic level, Se‐ or Le‐positive individuals carry at least one functional *FUT2* (*FUT2*  ^∗^ *01*) or *FUT3* (*FUT3*  ^∗^ *01*) allele, whereas Se‐ or Le‐negative individuals are homozygous for nonfunctional *se* (*FUT2*  ^∗^ *01N*) or *le* (*FUT3*  ^∗^ *01 N*) alleles [3, 5]. Of note, trace amounts of Le^a^ antigen are present in most individuals but are often below the threshold of routine phenotypic detection. Rarely, simultaneous expression of Le^a^ and Le^b^ may be observed, typically associated with reduced enzymatic efficiency of a functionally impaired *α*(1,2)‐FucT2 enzyme [6–8]. Mechanistically, the Le(a+b+) phenotype can arise in individuals with diminished secretor activity because of the c.418A>T variant in *FUT2*. The corresponding *FUT2*  ^∗^ *01W.02.01* (*Se^418^*) allele encodes an enzyme with markedly reduced activity (approximately 5% of wild‐type), which is sufficient to allow partial fucosylation and the resulting Le(a+b+) expression [8]. In addition to the c.418A>T variant, the Le(a+b+) phenotype may also be observed in individuals carrying other *FUT2*  ^∗^ *01W* alleles, including those with the *Se^w^*/*Se^w^* or *Se^w^*/*se* genotypes, as defined within the H blood group system [5].

The *FUT2* gene exhibits extensive allelic diversity with pronounced population specificity. In European, West Asian, and African populations, the nonsense variant c.461G>A (p.(Trp154Ter); *FUT2*  ^∗^ *01N.02*; *se^461^*) represents a predominant nonsecretor allele [9]. In East and Southeast Asian populations, the weak‐secretor allele c.418A>T (p.(Ile140Phe); *FUT2*  ^∗^ *01W.02.01*; *se^390,418^*) encodes a protein with reduced enzymatic activity rather than complete loss of function; the associated c.390C>T change is synonymous [8, 10]. Additional population‐restricted loss‐of‐function alleles include c.604C>T (p.(Arg202Ter); *FUT2*  ^∗^ *01N.04*; *se^390,604^*), which is common in Samoan and certain Taiwanese populations [11], c.882G>A (p.(Trp294Ter); *FUT2*  ^∗^ *01N.12*; *se^882^*) observed in certain Taiwanese populations [11], and c.335C>T (p.(Pro112Leu); *FUT2*  ^∗^ *01N.15*; *se^335^*) reported in Bangladeshi populations [12]. Functional *FUT2* alleles also display ethnic restriction. The synonymous variants c.73A>G (*FUT2*  ^∗^ *01.03.01*; *Se^73^*) and c.514G>A (*FUT2*  ^∗^ *01.06*; *Se^514^*) are predominantly found in African populations [13], whereas alleles such as *Se^408^* and *Se^433^* occur at high frequencies in New Guinean populations but are rare elsewhere [14]. In addition to single nucleotide variants (SNVs), structural alterations also contribute to *FUT2* diversity. Two alleles, *se^del^* and *se^del2^*, represent complete gene deletions generated by Alu–Alu recombination events [15, 16]. The *se^del^* allele was originally identified in individuals of Indian ancestry with the classic Bombay phenotype and has subsequently been reported at high frequency in Bangladeshi populations and among Sri Lankan groups (Sinhalese and Tamil) [15]. The *se^del2^* allele has been observed at high frequency in Samoan populations [16]. These genetic variations in *FUT2* and *FUT3* account for interpopulation differences in secretor status and the distribution of Le phenotypes.

The Le^a^ and Le^b^ antigens represent the clinically most relevant Le antigens and are routinely evaluated in transfusion settings; four principal Le phenotypes have been characterized. The distribution of Le(a+b−) and Le(a−b+) phenotypes varies among populations, with reported prevalences of 22% and 72% in Caucasians, 23% and 55% in Black populations, and 0.2% and 73% in Japanese populations, respectively [17]. In contrast, the Le(a+b+) phenotype remains rare in European and African populations but is comparatively frequent in East and Southeast Asia, the Pacific region, and Australasia, where reported prevalences range from 10% to 40% [18, 19]. Across three independent Thai blood donor cohorts, Le(a−b+) remained the predominant phenotype (51%–66%), followed by Le(a+b−) (11%–31%) and Le(a−b−) (18%–24%), with minimal temporal variation [20–22]. Although earlier studies did not detect the Le(a+b+) phenotype in Thai populations, these studies used either the conventional tube technique or gel‐based methods with polyclonal human anti‐Le^a^ and anti‐Le^b^ reagents. Thus, differences in phenotyping methods alone are unlikely to fully explain its absence. More recent observations, including our unpublished data, suggest a higher frequency of Le(a+b+) than previously reported. The discrepancy between earlier and more recent observations may reflect differences in population sampling or the distribution of underlying genetic determinants. A temporal change in population genetics, however, cannot be established from the available data.

Although the Le(a−b−) phenotype is the only Le phenotype capable of eliciting Le antibodies, its relatively higher frequency in Thailand contributes to the routine detection of Le antibodies in transfusion practice [4, 17]. Accordingly, accurate phenotypic characterization remains important for blood service management. Moreover, the Le(a+b+) phenotype, uncommon in many populations but comparatively frequent in Thailand, represents a biologically informative subgroup whose genetic basis has not been fully elucidated in this population. Previous studies have associated this phenotype with the c.418A>T variant and other *FUT2*  ^∗^ *01W* alleles but have not systematically examined the contribution of different *FUT2* genetic backgrounds and allele combinations in Thai individuals. In particular, the contribution of complete *FUT2* deletion alleles (*se^del^* or *se^del2^*) and their potential hemizygous configurations has not been systematically investigated in Thai or other Southeast Asian populations. These structural variants have been reported predominantly in South Asian and Samoan populations, and their distribution and phenotypic contribution in Southeast Asian populations remain insufficiently defined. Therefore, this study is aimed at characterizing the *FUT2* and *FUT3* variation across Le phenotypes in Thai blood donors and determine the contribution of weakened, nonfunctional, and deletion alleles, together with their zygosity and allele combinations, to the molecular basis of the Le(a+b+) phenotype.

## 2. Materials and Methods

### 2.1. Study Population

Between November 2025 and January 2026, a total of 708 first‐time blood donors were recruited from the Blood Bank Unit, Thammasat University Hospital (TUH), following the provision of written informed consent. Peripheral blood samples anticoagulated with ethylenediaminetetraacetic acid (EDTA) were collected from all participants and subsequently subjected to serological analyses, including Le blood group phenotyping. Consequently, the study population was divided into two cohorts. The first cohort comprised the first 151 consecutively recruited donors, in whom all Le phenotypes [Le(a+b−), Le(a−b+), Le(a+b+), and Le(a−b−)] were included. The second cohort consisted of the remaining 557 donors, from whom only individuals exhibiting the Le(a+b+) phenotype were selected. A total of 100 Le(a+b+) donors from this cohort were subsequently enrolled for further analyzes. Demographic and serological data, including age, sex, and ABO and Rh blood groups, were obtained from the TUH donor database.

The study was conducted in accordance with the principles of the Declaration of Helsinki and approved by the Committee on Human Rights Related to Research Involving Human Subjects, Thammasat University, Pathum Thani, Thailand (COE No. 033/2568). Written informed consent was obtained from all participants prior to enrollment. The informed consent included permission for genetic analyses of blood group systems, including the *FUT2* and *FUT3* blood group genes, and the use of participants′ blood samples for genetic research.

### 2.2. Le Blood Group Phenotyping

Le phenotyping was performed using DiaClon Anti‐Le^a^ and Anti‐Le^b^ gel cards (BIO‐RAD, Cressier, Switzerland). Briefly, a 5% RBC suspension prepared in Diluent‐I (BIO‐RAD, Cressier, Switzerland) was incubated at room temperature (18°C–25°C), loaded into the corresponding microtubes, and centrifuged according to the manufacturer′s protocol. Agglutination reactions were graded from 4+ to 0, with reaction strengths ranging from 4+ to w+ considered positive and a reaction grade of 0 considered negative for the corresponding Le antigen. Agglutination patterns were interpreted and recorded following the manufacturer′s instructions.

### 2.3. DNA Extraction

Genomic DNA was extracted from peripheral whole blood using the QIAamp DNA Blood Kit (Qiagen, Hilden, Germany) according to the manufacturer′s instructions. A total of 251 donor samples were stratified into two study groups for downstream genetic analysis. DNA samples were stored at −20°C until further analysis.

### 2.4. PCR Amplification, DNA Sequencing, and Variant Analysis

The reference genomic and transcript sequences of *FUT2* (NG_007511.1 and NM_000511.6) and *FUT3* (NG_007482.2 and NM_000149.4), encoding proteins of 343 and 361 amino acids, respectively, were used for sequence alignment and variant calling. Sequence variants were described according to the Human Genome Variation Society (HGVS) recommendations using the respective reference transcript sequences. PCR amplification was performed using the primer pairs listed in Table 1, and amplicons were subjected to bidirectional Sanger sequencing as previously described [23, 24]. The coding regions of *FUT2* and *FUT3* were amplified by PCR. Each PCR was performed in a final reaction volume of 40 *μ*L containing 3 *μ*L of genomic DNA (50 ng/*μ*L), 3 *μ*L each of forward and reverse primers, 20 *μ*L of 2x Phusion High‐Fidelity PCR Master Mix (New England BioLabs, Ipswich, Massachusetts, United States), and 11 *μ*L of nuclease‐free water. Amplification was carried out using a T100 Thermal Cycler (Bio‐Rad, Hercules, California, United States). Target genomic regions were amplified by PCR under the following cycling conditions: initial denaturation at 98°C for 1 min; 10 cycles of denaturation at 98°C for 30 s and annealing at 69°C for 60 s; followed by 30 cycles of denaturation at 98°C for 10 s, annealing at 63°C for 50 s, and extension at 72°C for 30 s; and a final extension at 72°C for 5 min. This protocol generated amplicons of 1188 bp (*FUT2*), 869 bp (*FUT3*‐EX3‐A1), and 1042 bp (*FUT3*‐EX3‐A2), respectively.

**Table 1:** Oligonucleotide primers used for PCR amplification and sequencing.

Primer name	Sequence (5 ^′^to 3 ^′^)	Gene	Amplicon size (bp)	Final concentration (*μ*mol/L)
FUT2‐F [23]	CCATCTCCCAGCTAACGTGTCC	*FUT2*	1188	0.75
FUT2‐R [23]	GGGAGGCAGAGAAGGAGAAAAGG			0.75
LEW‐EX3‐A1‐F [24]	GCAGCTCCTCTCAGGACTCA	*FUT3*	869	0.75
LEW‐EX3‐A1‐R [24]	TGATGTAGTCGGGGTGCAAG			0.75
LEW‐EX3‐A2‐F [24]	CCAAGGGGACCATGATGGAGAC		1042	0.75
LEW‐EX3‐A2‐R [24]	AGTCGATCCCACCTGTACCCTA			0.75
HGH‐F	TGCCTTCCCAACCATTCCCTTA	*HGH*	434	0.5
HGH‐R	CCACTCACGGATTTCTGTTGTGTTTC			0.5
Bombay‐U‐nest [16]	GCGGAGAGCTGGGTTATTTCACGGGAACAG	Intron and downstream of the 3 ^′^ breakpoint of *FUT2*	1800 or 2600	0.5
Bombay‐L [16]	GTGTCTCGGGCACTCGTCTTTCAGC			0.5

PCR products were separated by electrophoresis on a 1.5% agarose gel containing SYBR Safe DNA Gel Stain (Invitrogen, Paisley, United Kingdom) in 1x TBE buffer at 100 V. DNA fragments were visualized using a blue‐light transilluminator. The target amplicons were excised and purified using the GeneJET Gel Extraction Kit (Thermo Scientific, Waltham, Massachusetts, United States), and the purified products were sequenced bidirectionally (Sanger Sequencing Services, U2bio (Thailand) Co., Ltd., Bangkok, Thailand) using the same PCR primers. Allelic phase resolution for heterozygous variants was performed using next‐generation sequencing (NGS) on the Illumina platform (FastNGS Sequencing Services, U2bio (Thailand) Co., Ltd., Bangkok, Thailand). A minimum sequencing depth of 100x and at least 10 phase‐informative reads were required for haplotype assignment. Each phased BAM and its corresponding BAI index file were manually reviewed to confirm the presence of both alleles at each variant position. Regions with insufficient phase‐informative reads were considered unphased and were not assigned by inference.

### 2.5. *se^del^* and *se^del2^* Genotyping Identification

Two distinct Alu‐mediated deletions of the *FUT2* allele were analyzed using 10 *μ*L PCR reaction mixtures containing 2x PCR Master Mix (i‐StarMAXII, iNtRON Biotechnology, Gyeonggi‐do, Korea), allele‐specific primer pairs, 50–150 ng of genomic DNA, and HGH internal control primers. PCR was performed using the same primers as previously described [16]; final primer concentrations are shown in Table 1. Amplification was carried out on a T100 Thermal Cycler (Bio‐Rad, Hercules, California, United States). Thermal cycling comprised an initial denaturation at 94°C for 4 min, followed by 35 cycles of denaturation at 94°C for 15 s, annealing at 69°C for 40 s, and extension at 72°C for 2 min, with a final extension at 72°C for 5 min. PCR products were resolved on 1.5% agarose gels in 1x TBE buffer containing SYBR Safe DNA Gel Stain and visualized under blue light at 100 V. The expected amplicons corresponding to *se^del^* (1.8 kb) and *se^del2^* (2.6 kb) were identified accordingly.

### 2.6. Statistical Analysis

Phenotype frequencies were summarized using descriptive statistics and expressed as percentages. Genotype and allele frequencies were determined by direct counting. Continuous variables with a normal distribution were expressed as mean ± standard deviation (SD), whereas nonnormally distributed variables were expressed as median and interquartile range (IQR). Observed genotype frequencies were assessed for deviation from Hardy–Weinberg equilibrium (HWE) using the chi‐square (*χ* ^2^) test. Differences in allele frequency distributions between the Thai population and other populations were evaluated using the *χ* ^2^ test of homogeneity. When a variant *FUT2* allele was identified in the homozygous, heterozygous, or hemizygous state, the corresponding Le phenotypes were compared. All data cleaning and statistical analyses were performed using SPSS Statistics, Version 25.0 software (IBM Corp., Armonk, New York, United States), with the threshold for statistical significance set at *p* ≤ 0.05.

## 3. Results

### 3.1. Participant Characteristics and Le Phenotype Distribution

Among the first 151 consecutively recruited donors, all Le phenotypes were included for subsequent molecular analysis. Le(a−b+) was the predominant Le phenotype, accounting for 87 (57.62%) donors, followed by Le(a+b+) in 36 (23.84%), Le(a−b−) in 24 (15.89%), and Le(a+b−) in four (2.65%). The ABO/RhD blood group distribution comprised O, RhD‐positive in 57 (37.75%) donors; B, RhD‐positive in 48 (31.79%); A, RhD‐positive in 31 (20.53%); AB, RhD‐positive in 14 (9.27%); and B, RhD‐negative in one (0.66%). The cohort included 68 male and 83 female donors, with a mean age of 35.0 ± 11.6 years (range, 18–61 years).

The second cohort comprised the remaining 557 donors, of whom 100 with the Le(a+b+) phenotype (17.95%) were selected for subsequent molecular analysis. The Le phenotype distribution was similar, with Le(a−b+) in 313 (56.19%) donors, Le(a−b−) in 132 (23.70%), Le(a+b+) in 100 (17.95%), and Le(a+b−) in 12 (2.15%). The ABO/RhD blood group distribution comprised O, RhD‐positive in 208 (37.34%) donors; B, RhD‐positive in 180 (32.32%); A, RhD‐positive in 120 (21.54%); AB, RhD‐positive in 47 (8.44%); O, RhD‐negative in 1 (0.18%); and AB, RhD‐negative in 1 (0.18%). The cohort included 250 male and 307 female donors, with a mean age of 35.4 ± 13.1 years (range, 18–64 years).

### 3.2. Two Distinct *FUT2* Deletion Alleles

To investigate structural variation in *FUT2*, the coding region was amplified and sequenced in all 151 donors from the first cohort, representing all Le phenotypes, and in the 100 selected Le(a+b+) donors from the second cohort. PCR amplification was successful for all samples. Three samples showed amplification patterns suggestive of *se^del^* or *se^del2^* deletion alleles during genotyping. Direct Sanger sequencing of these samples subsequently showed homozygous nucleotide variant patterns, which, together with the genotyping results, were interpreted as hemizygous sequence patterns. Deletion‐specific PCR assays identified a 1.8‐kb amplicon corresponding to the *se^del^* allele in two donors (2/208, 0.96%; one from each cohort) and a 2.6‐kb amplicon corresponding to the *se^del2^* allele in one donor (1/208, 0.48%) from the second cohort (Figure 1). The deletion‐specific PCR products were subsequently confirmed by NGS. NGS read alignment confirmed the deletion breakpoint junctions in all three donors, as shown by representative Integrative Genomics Viewer (IGV) plots (Figure S1). The flanking sequences spanning the deletion junctions from LEW062 (*se^del^*), LEW163 (*se^del2^*), and LEW234 (*se^del^*) were submitted to GenBank and assigned accession numbers QB065495, QB058350, and QB065494, respectively. Sequence alignment confirmed the junctions of both the *se^del^* and *se^del2^* alleles, demonstrating recombination between the Alu elements at the 5 ^′^ and 3 ^′^ breakpoints. Additional *FUT1* sequence analysis was performed in the *FUT2*‐deleted samples. Neither the c.725T>G variant, previously reported in association with *FUT2* deletion alleles, nor any other inactivating *FUT1* variants were identified. These findings demonstrate the presence of two distinct *FUT2* deletion alleles (*se^del^* and *se^del2^*) in the Thai population.

**Figure 1:**
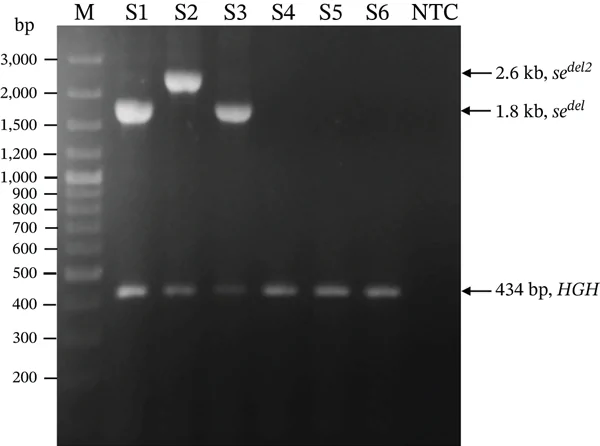
Modified PCR assay for detection of the *se^del^* and *se^del2^* junction regions. PCR products were separated by electrophoresis on a 1.5% agarose gel containing SYBR Safe DNA Gel Stain. The 434‐bp *HGH* internal control amplicon was detected in all lanes, confirming successful PCR amplification. Lanes S1 and S3 show a donor heterozygous for *se^del^* (1.8 kb), whereas lane S2 shows donors heterozygous for *se^del2^* (2.6 kb). Lanes S4–S6 represent control individuals. Lane M contains a GeneRuler 100 bp Plus DNA Ladder used as the molecular size marker.

### 3.3. Distribution of *FUT2* and *FUT3* Variants in the Thai Population

The coding regions of *FUT3* and *FUT2* were amplified and sequenced in all 151 donors from the first cohort. Analysis of the *FUT2* and *FUT3* coding sequences identified 18 SNVs in *FUT2* and six SNVs in *FUT3*. The genotype frequencies of each SNV are shown in Figure 2a,b. Among the *FUT2* variants, c.390C>T and c.418A>T were the most frequent, with homozygous wild‐type, heterozygous, and homozygous variant genotype frequencies of 0.040, 0.252, and 0.709 for c.390C>T, and 0.305, 0.444, and 0.252 for c.418A>T, respectively. The remaining *FUT2* variants occurred at lower frequencies, with homozygous variant frequencies ranging from 0.000 to 0.020. Among the *FUT3* variants, c.59T>G and c.1067T>A were the most frequent. For c.59T>G, the genotype frequencies of TT, TG, and GG were 0.371, 0.470, and 0.159, respectively, whereas those of c.1067T>A (TT, TA, and AA) were 0.570, 0.278, and 0.152, respectively, whereas the remaining variants exhibited homozygous variant frequencies of ≤ 0.013. All observed SNV genotype frequencies conformed to HWE, except for *FUT2* c.335C>T, c.461G>A, c.642G>A, c.772G>A, and c.993A>G, and *FUT3* c.1067T>A, which showed significant deviations from HWE (*χ* ^2^ = 65.76, 66.44, 151.00, 28.88, 15.45, and 16.09, respectively; *p* = 5.10 × 10^−16^, 3.61 × 10^−16^, 1.05 × 10^−34^, 7.71 × 10^−8^, 8.49 × 10^−5^, and 6.03 × 10^−5^, respectively).

**Figure 2:**
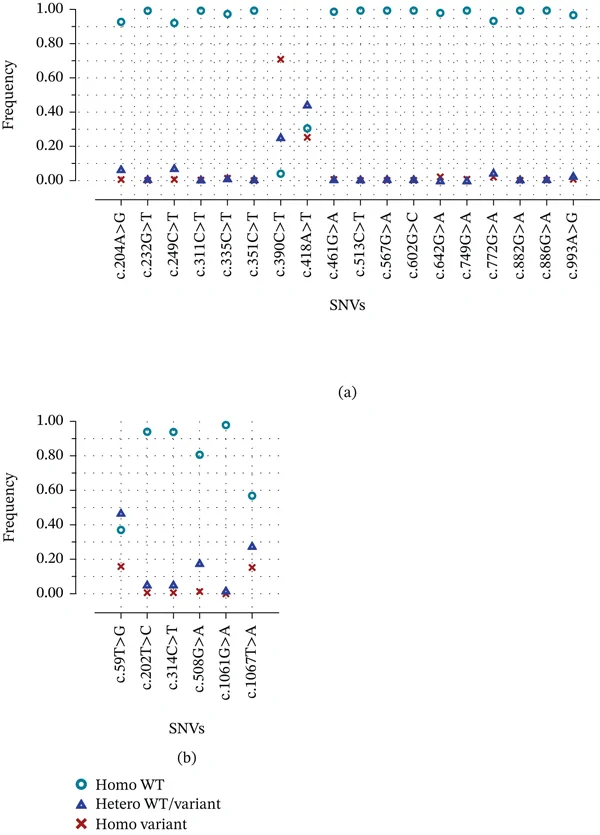
Genotype frequencies of *FUT2* and *FUT3* SNVs in the first cohort of 151 Thai blood donors. (a) Frequencies of the 18 *FUT2* SNVs. (b) Frequencies of the six *FUT3* SNVs. Open circles denote homozygous wild‐type (homo WT), open triangles denote heterozygous (WT/variant), and red crosses denote homozygous variant (homo variant) genotypes.

### 3.4. Haplotype Analysis and *FUT2–FUT3* Genotype Combinations

Among the 151 donors in the first cohort, the distribution and functional classification of *FUT2* and *FUT3* alleles are summarized in Table 2. The nomenclature and corresponding HGVS and ISBT designations, where available, for the *FUT2* and *FUT3* alleles identified in this study are provided in Table S1. A total of 26 distinct *FUT2* alleles were identified. Within the functional class, *Se^390^* was predominant (102/302, 0.338), followed by *Se* (26/302, 0.086). The reduced‐function class was dominated by *Se^390,418^* (132/302, 0.437), whereas the remaining alleles occurred at frequencies of 0.003–0.013. Multiple loss‐of‐function *FUT2* alleles were observed, with *se^204,249,772^* and *se^204,249,772,993^* being the most frequent (4/302 each, 0.013). The *se^del^* allele occurred at a frequency of 0.003 (1/302). For *FUT3*, 10 distinct alleles were identified. *Le* was the predominant functional allele (155/302, 0.513), followed by *Le^1067^* (17/302, 0.056). *Le^59^* was the predominant reduced‐function allele (30/302, 0.099), whereas *le^59,1067^* was the most frequent loss‐of‐function allele (57/302, 0.189), followed by *le^59,508^* (20/302, 0.066), *le^59,508,1067^* (10/302, 0.033), and *le^202,314^* (9/302, 0.030). The remaining loss‐of‐function *FUT3* alleles occurred at frequencies of 0.003–0.007.

**Table 2:** Distribution and functional classification of *FUT2* and *FUT3* alleles in the Thai population (*N* = 151).

Gene	Functional class	Allele	*N*(frequency)	Gene	Functional class	Allele	*N*(frequency)
*FUT2*	Functional	*Se^390^*	102 (0.338)	*FUT3*	Functional	*Le*	155 (0.513)
		*Se*	26 (0.086)			*Le^1067^*	17 (0.056)
		*Se^390,513^*	4 (0.013)		Reduced‐function	*Le^59^*	30 (0.099)
		*Se^351,390^*	2 (0.007)		Loss‐of‐function	*le^59,1067^*	57 (0.189)
		*Se^204,249^*	1 (0.003)			*le^59,508^*	20 (0.066)
		*Se^204,249,642^*	1 (0.003)			*le^59,508,1067^*	10 (0.033)
		*Se^390,749^*	1 (0.003)			*le^202,314^*	9 (0.030)
	Reduced‐function	*Se^390,418^*	132 (0.437)			*le^59,508,1061^*	2 (0.007)
		*Se^390,418,642^*	4 (0.013)			*le^202,314,1067^*	1 (0.003)
		*Se^418^*	3 (0.010)			*le^59,1061^*	1 (0.003)
		*Se^311,390,418^*	1 (0.003)				
		*Se^390,418,567^*	1 (0.003)				
		*Se^390,418,749^*	1 (0.003)				
		*Se^886^*	1 (0.003)				
	Loss‐of‐function	*se^204,249,772^*	4 (0.013)				
		*se^204,249,772,993^*	4 (0.013)				
		*se^335^*	3 (0.010)				
		*se^204,249,461,772,993^*	2 (0.007)				
		*se^772^*	2 (0.007)				
		*se^204,249,335^*	1 (0.003)				
		*se^232^*	1 (0.003)				
		*se^390,418,461^*	1 (0.003)				
		*se^390,602^*	1 (0.003)				
		*se^390,772^*	1 (0.003)				
		*se^882^*	1 (0.003)				
		*se^del^*	1 (0.003)				

*Note:* Allele frequencies were calculated based on 302 alleles from 151 donors (2 × 151). *FUT2* and *FUT3* alleles were classified as functional, reduced‐function, or loss‐of‐function according to the expected functional activity of the encoded *α*(1,2)‐FucT2 and *α*(1,3/1,4)‐FucT3, respectively.

Abbreviations: *Se* and *Le*, functional alleles; *se* and *le*, nonfunctional alleles.

Haplotype analysis demonstrated substantial diversity in the combined *FUT2*–*FUT3* genotypes associated with the observed Le phenotypes and their categorized agglutination strengths (Table S2). The Le(a−b+) phenotype was predominantly observed among donors carrying secretor *FUT2* genotypes in combination with functional *FUT3* alleles. In contrast, Le(a+b+) was frequently observed in the presence of the reduced‐function *FUT2*, *Se^390,418^* haplotype. These genotypes commonly carried a functional *FUT3*, *Le* allele together with diverse second *FUT3* alleles, spanning functional, reduced‐function, and loss‐of‐function variants. The Le(a−b−) phenotype occurred across secretor, weak‐secretor, and nonsecretor *FUT2* backgrounds, with all individuals carrying *FUT3* loss‐of‐function alleles. The Le(a+b−) phenotype was observed in association with nonsecretor *FUT2* genotypes and functional *FUT3* alleles.

The distribution of Le phenotypes across combined *FUT2–FUT3* genotype configurations among the 151 donors in the study cohort is shown in Figure 3. Le(a−b+) predominated among functional *FUT2* genotypes, particularly *Se*/*Se* and *Se*/*Se^w^*, in combination with functional *FUT3* genotypes. Le(a+b+) was most frequently observed with the weak‐secretor *Se^w^*/*Se^w^* genotype, particularly in combination with *Le*/*Le* (*N* = 19) and *Le*/*le* (*N* = 7). Le(a−b−) occurred across multiple *FUT2–FUT3* configurations, with the largest groups observed for *Se*/*Se^w^*–*le*/*le* (*N* = 10) and *Se^w^*/*Se^w^*–*le*/*le* (*N* = 8). Le(a+b−) was restricted to the *se*/*se* genotype and occurred with *Le*/*Le* or *Le*/*le* (*N* = 2 each). The predicted Le phenotypes based on the combined *FUT2–FUT3* genotype configurations were concordant with the observed serological phenotypes in all 151 donors, corresponding to 100% concordance and 100% overall prediction accuracy.

**Figure 3:**
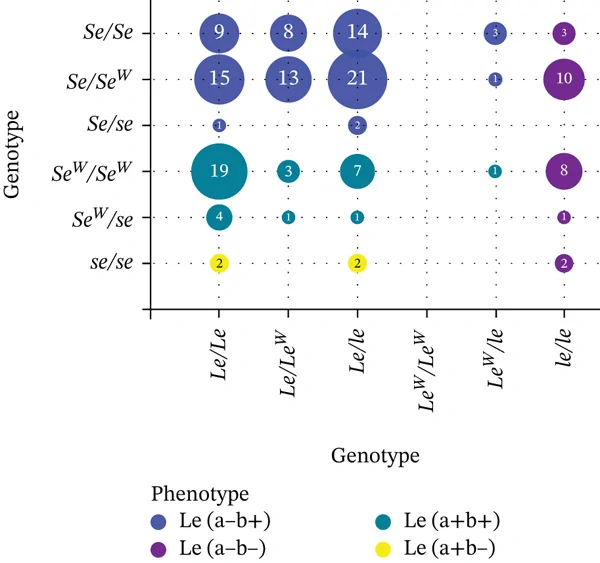
Le phenotype patterns determined by column agglutination testing (CAT) according to combined *FUT2–FUT3* genotypes in the study cohort. Circle size represents the number of samples; numbers indicate the sample size (*N*) for each genotype. *Se* and *Le* denote functional alleles, whereas *se* and *le* denote nonfunctional alleles. *Se^w^* and *Le^w^* denote weakened alleles.

### 3.5. *FUT2* Variant Combinations Underlying the Le(a+b+) Phenotype

To further characterize the genetic basis of the Le(a+b+) phenotype, *FUT2* variant combinations were analyzed in an additional cohort of 100 donors with the Le(a+b+) phenotype (Table 3). Notably, all 100 donors carried the weakened *Se^390,418^* allele, either in the homozygous state or in combination with a deleted, nonfunctional, or functionally uncertain *FUT2* allele. The majority of donors (73/100) were homozygous for *Se^390,418^*. Two distinct hemizygous configurations involving *Se^390,418^* with either the *se^del^* or *se^del2^* deletion were identified, each in one donor. The remaining 25 donors carried compound heterozygous *Se^390,418^*/nonfunctional *FUT2* combinations, representing compensated weakened/null configurations. Among these, *Se^390,418^/se^335^* was the most frequent combination (8/100), followed by *Se^390,418^/se^204,249,772^* (7/100), *Se^390,418^/se^390,604^* (4/100), *Se^390,418^/se^204,249,461^* (2/100), *Se^390,418^/se^882^* (2/100), and *Se^390,418^/se^390,901^* (1/100). One donor carried the rare novel *FUT2* variant *Se(se)^676^*, which was categorized as having an uncertain weakened/null status based on the available evidence.

**Table 3:** Distribution and functional classification of *FUT2* variant combinations identified in donors with the Le(a+b+) phenotype (*N* = 100).

**Nucleotide position**		**204**	**249**	**335**	**390**	**418**	**461**	**604**	**676**	**772**	**882**	**901**	***se*** ^***del***^	***se*** ^***del2***^	**Number of subjects**
**Nucleotide**		**A**	**C**	**C**	**C**	**A**	**G**	**C**	**G**	**G**	**G**	**G**			
**Amino acid**		**Ala**	**Tyr**	**Pro**	**Asn**	**Ile**	**Trp**	**Arg**	**Ala**	**Gly**	**Trp**	**Gly**			
Homozygous weakened alleles															
*Se^390,418^*	*Se^390,418^*	.	.	.	T	T	.	.	.	.	.	.	ND	ND	73
Hemizygous															
*Se^390,418^*	*se^del^*	.	.	.	T	T	.	.	.	.	.	.	D	ND	1
*Se^390,418^*	*se^del2^*	.	.	.	T	T	.	.	.	.	.	.	ND	D	1
Compensated heterozygous weakened/null alleles															
*Se^390,418^*	*se^335^*	.	.	C/T	C/T	A/T	.	.	.	.	.	.	ND	ND	8
*Se^390,418^*	*se^204,249,772^*	A/G	C/T	.	C/T	A/T	.	.	.	G/A	.	.	ND	ND	7
*Se^390,418^*	*se^390,604^*	.	.	.	T	A/T	.	C/T	.	.	.	.	ND	ND	4
*Se^390,418^*	*se^204,249,461^*	A/G	C/T	.	C/T	A/T	G/A	.	.	.	.	.	ND	ND	2
*Se^390,418^*	*se^882^*	.	.	.	C/T	A/T	.	.	.	.	G/A	.	ND	ND	2
*Se^390,418^*	*se^390,901^*	.	.	.	T	A/T	.	.	.	.	.	G/A	ND	ND	1
Uncertain weakened/null classification															
*Se^390,418^*	***Se(se)*** ^***676***^	.	.	.	C/T	A/T	.	.	G/**A**	.	.	.	ND	ND	1
**Nucleotide change**		G	T	T	T	T	A	T	**A**	A	A	A			
**Amino acid position**		68	83	112	130	140	154	202	**226**	258	294	301			
**Amino acid change**		=	=	Leu	=	Phe	Ter	Ter	**Thr**	Ser	Ter	Arg			

*Note:* The sequences NG_007511.1 and NM_000511.6 were used as references for the nucleotide sequence and base numbering, respectively. Novel sequence variations, their nucleotide positions, and the corresponding novel alleles (second column) are indicated in boldface. When two or more heterozygous polymorphisms were identified within the examined region, haplotypes were characterized by next‐generation sequencing; the secretor status and nucleotide bases located on the same chromosome are underlined. Bases identical to the reference sequence are indicated by “.” in the table. Synonymous substitutions are indicated as “=,” and “Ter” denotes a stop codon in the amino acid change column. The underlined nucleotide bases indicate positions located on the same chromosome (in cis).

Abbreviations: D, detected deletion; ND, not detected; *Se* and *Le*, functional alleles; *se* and *le*, nonfunctional alleles; *Se(se)*, alleles with an ambiguous functional classification for which it could not be definitively determined whether they were functional (*Se*) or nonfunctional (*se*).

The missense variant c.676G>A (p.(Ala226Thr); rs1410377195) was rare in population databases, with an allele frequency of 8.06 × 10^−6^ (13/1,613,590 alleles) in gnomAD v4.1.1, with no homozygous individuals reported [25]. No ClinVar record was identified for this variant. The variant is located within the lumenal region of *α*1,2FucT2 (residues 29–343). Structural annotations from ProtVar (Version 1.4) indicated that the variant position was not located within a predicted protein pocket or protein–protein interaction site [26]. In silico predictions were discordant. Despite a high conservation score (0.96), CADD and ESM‐1b predicted a potentially deleterious effect, whereas FoldX predicted reduced protein stability. In contrast, AlphaMissense, popEVE, and Missense3D yielded ambiguous, likely nondeleterious, and neutral predictions, respectively. PolyPhen‐2 (Polymorphism Phenotyping, Version 2.2.2) classified the variant as possibly damaging, with a score of 0.785 (sensitivity, 0.85; specificity, 0.93) [27]. The variant was confirmed by Sanger sequencing, as shown in the electropherogram in Figure S2.

## 4. Discussion

In this study, we investigated the genetic basis of the relatively high prevalence of the Le(a+b+) phenotype in the Thai population. Our findings reveal a complex genetic architecture involving heterogeneous *FUT2* allele combinations, including functional, reduced‐function, and loss‐of‐function alleles, across diverse *FUT3* genetic backgrounds. These findings suggest that Le(a+b+) is not attributable to a single *FUT2* variant but may arise from multiple allelic configurations associated with varying levels of secretor activity, potentially contributing to differences in Le phenotype distributions among Thai populations [20–22]. The phenotype distribution in our cohorts was consistent with previous Thai studies, in which Le(a−b+) predominated and Le(a+b+) was the second most frequent phenotype [20–22, 28]. The observed Le(a+b+) frequency of 23.84% also falls within the reported range for East and Southeast Asian populations (approximately 10%–40%) [18, 19] and is comparable to the 24.22% reported among blood donors in Northern Vietnam [29]. In the Philippines, multiple *FUT2* genotype combinations have been identified among individuals with Le(a+b+) [30], suggesting that genetic heterogeneity may also contribute to Le phenotype variation in other Southeast Asian populations. However, differences in study populations, serological methods, and genotyping approaches limit direct comparisons. These regional patterns raise the possibility that specific combinations of *FUT2* and *FUT3* alleles contribute to the relatively high prevalence of Le(a+b+) in Thailand.

Rather than being explained by a single predominant *FUT2* allele, the Thai cohort demonstrated a heterogeneous spectrum of *FUT2* sequence variation, accompanied by multiple *FUT3* variants. In the first cohort, sequencing identified 18 SNVs in *FUT2* and six SNVs in *FUT3*, indicating substantial allelic heterogeneity among individuals with different Le phenotypes. Most SNVs showed genotype distributions consistent with HWE. However, significant deviations from HWE were observed for *FUT2* c.335C>T, c.461G>A, c.642G>A, c.772G>A, and c.993A>G, and for *FUT3* c.1067T>A. These variants occurred at relatively low frequencies, with several genotype classes represented by few individuals. Although sampling variation and the limited representation of rare genotype classes may contribute to these deviations, other factors, including population stratification, genotyping error, or selection, cannot be excluded. Therefore, the observed HWE deviations should be interpreted cautiously and should not be considered evidence of population‐level departures from HWE. Larger and more representative Thai population cohorts will be necessary to determine whether these deviations are reproducible.

Among the *FUT2* variants, c.390C>T and c.418A>T were the most frequent. The synonymous c.390C>T variant, formerly designated c.357C>T, has no apparent effect on protein function and was detected in 145 of 151 donors (96.0%) in our first cohort. Its high prevalence is consistent with the allele frequencies reported in Asian populations, including Vietnamese (87.2%) [31], Thai (88.9%), Indonesian (89.3%–92.9%), Taiwanese Han (89.2%), Japanese (75.0%), Filipino (85.5%), and Indigenous Taiwanese populations (60.5%–98.5%) [32]. The c.418A>T variant had a T‐allele frequency of 47.4% in our cohort, consistent with reported frequencies in Vietnamese (59.0%) [31], Thai (48.1%–50.9%), Indonesian (48.5%–63.1%), Taiwanese Han (47.4%), and Japanese (40.7%) populations [32], as well as the approximately 44% reported for East Asians in gnomAD v4.1.1 [25]. Thus, both variants reflect the broader distribution of common *FUT2* variation in Asian populations [18, 19]. However, their high frequencies alone do not explain the relatively high prevalence of Le(a+b+), suggesting that additional *FUT2* variants and their allelic combinations contribute to the genetic architecture of this phenotype.

Among the *FUT3* variants, c.59T>G and c.1067T>A were the most frequent in our cohort, with variant‐allele frequencies of 39.4% and 29.1%, respectively. Although c.59T>G is widely distributed in Asian populations, its contribution to the Le(a−b−) phenotype could not be assessed because only one individual with this phenotype was identified; this individual was homozygous for *le^202,314^* rather than carrying c.59T>G. This contrasts with a previous Thai study in which c.59T>G was detected in all individuals with Le(a−b−), alongside c.1067T>A and c.508G>A frequencies of 87.5% and 35.7%, respectively [24]. These differences may reflect variation in the *FUT3* allelic backgrounds underlying this phenotype, although the small number of Le(a−b−) individuals in our cohort precludes further interpretation. The c.59T>G allele has also been reported in northern and eastern Chinese populations at frequencies of 43.13% and 39.87%, respectively, across individuals with different Le phenotypes [33, 34]. Population‐level data further demonstrate its distribution across African (33.7%), American (27.1%), European (9.8%), South Asian (24.0%), and East Asian (29.6%) populations [33]. Overall, these findings indicate that common *FUT3* variants are widely distributed, but their associations with specific Le phenotypes may depend on the underlying allelic background.

In addition to common functional and weak‐secretor alleles, we identified two distinct large *FUT2* deletions, *se^del^* and *se^del2^*. Although rare in our cohort, both were confirmed by NGS and have been reported in geographically distinct populations, with *se^del^* reported at an allele frequency of 0.074 in Bangladesh and *se^del2^* at 0.104 in Samoa [16]. These findings indicate that large *FUT2* deletions occur across diverse populations and may contribute to the genetic basis of *FUT2* variation. The identification of *se^del^* carriers also has implications for the molecular interpretation of Bombay and para‐Bombay phenotypes. Previous studies have linked *se^del^* and the *FUT1* c.725T>G variant to the Bombay phenotype [15]; however, the occurrence of *se^del^* without c.725T>G in Bangladesh and in our cohort indicates that these variants are not necessarily linked. Thus, detection of an unexpected H‐antigen phenotype may warrant additional *FUT2* deletion analysis when routine *FUT1* findings are insufficient to explain the serologic phenotype. Because conventional Sanger sequencing may not reliably detect heterozygous large deletions, deletion‐specific PCR or other structural‐variant approaches may be useful in selected cases.

Importantly, Le antigen expression cannot be interpreted solely on the basis of *FUT2* genotype. Le antigen biosynthesis depends on the coordinated activities of *α*(1,2)‐FucT2 and *α*(1,3/1,4)‐FucT3, such that consideration of both *FUT2*‐secretor activity and the underlying *FUT3* genotype provides a more informative framework for interpreting Le phenotypes [4]. In our cohort, Le(a−b+) was predominantly associated with functional *FUT2* and *FUT3* backgrounds, consistent with efficient formation of type‐1 H and subsequent Le^b^ synthesis. In contrast, Le(a+b+) was most frequently observed among individuals with the reduced‐function *Se^w^/Se^w^* genotype, particularly in combination with functional *FUT3* alleles. This pattern is consistent with the established role of reduced *α*(1,2)‐FucT2 activity in limiting conversion of Type‐1 precursor structures toward Le^b^, thereby permitting the coexistence of Le^a^ and Le^b^ determinants [4, 6]. Importantly, however, *Se^w^/Se^w^* was observed with more than one *FUT3* configuration, including *Le/Le* and *Le/le*, indicating that the relatively high frequency of Le(a+b+) in the Thai population is not attributable to a single *FUT2–FUT3* diplotype but rather reflects multiple genetic configurations involving reduced secretor activity and functional *FUT3* backgrounds.

The Le(a−b−) phenotype further illustrates the contribution of *FUT3* variation to Le antigen expression. Although observed across different *FUT2* functional backgrounds, all individuals with the Le(a−b−) phenotype carried *FUT3* loss‐of‐function alleles, consistent with the requirement for functional *α*(1,3/1,4)‐FucT3 activity for the synthesis of both Le^a^ and Le^b^ [4, 24]. Conversely, Le(a+b−) was observed in association with non‐secretor *FUT2* genotypes and functional *FUT3* alleles, consistent with the established biosynthetic pathway in which functional *α*(1,3/1,4)‐FucT3 converts Type‐1 precursor structures to Le^a^ in the absence of *α*(1,2)‐FucT2*-*mediated formation of Type‐1 H [4, 5]. Overall, the distribution of Le phenotypes in our cohort was broadly consistent with the established biochemical model of Le antigen biosynthesis while revealing substantial genetic heterogeneity at both the *FUT2* and *FUT3* loci. These findings indicate that Le phenotypes in the Thai population reflect the combined effects of *FUT2*‐secretor activity, *FUT3* function, and the specific allelic combinations present on both loci. This multilocus genetic architecture provides a plausible genetic explanation for the relatively high frequency of Le(a+b+) observed in Thais.

Given the contribution of both *FUT2* and *FUT3* to Le phenotypes, we further focused on *FUT2* variation in an independent Le(a+b+) cohort to identify variant combinations associated with this phenotype. This analysis provided additional evidence for a heterogeneous *FUT2* genetic architecture centered on the weakened *Se^390,418^* allele. All 100 individuals carried *Se^390,418^*, either in the homozygous state or in combination with a nonfunctional, deleted, or functionally uncertain allele. The predominance of the *Se^390,418^/Se^390,418^* genotype suggests that this weakened‐secretor allele represents a major genetic background associated with the Le(a+b+) phenotype in the Thai population. Previous functional studies have estimated that the *Se^390,418^* allele retains approximately 5% of wild‐type *α*(1,2)‐FucT2 activity [7]. Although the present study did not directly measure enzyme activity, our genotype–phenotype findings provide indirect evidence that this residual function may be sufficient to support Le^b^ expression. In particular, Le(a+b+) was observed not only in individuals with the *Se^390,418^/Se^390,418^* genotype but also in individuals carrying *Se^390,418^* in combination with *se^del^*, *se^del2^*, or *se* ^335^. These findings suggest that the residual *α*(1,2)‐FucT2 function associated with a single *Se^390,418^* allele may be sufficient for Le^b^ synthesis despite reduced or complete loss of function of the other allele. This pattern is consistent with a threshold model in which Le^b^ expression is maintained when residual *α*(1,2)‐FucT2 function exceeds a minimum functional threshold. However, the present data do not establish the quantitative activity threshold, and Le^b^ expression may also depend on the *FUT3* genetic background and the availability of Type‐1 precursor structures. Thus, the phenotypic consequences of *FUT2* variation may reflect quantitative differences in residual *α*(1,2)‐FucT2 function as well as the specific combination of alleles, rather than a simple functional versus nonfunctional classification [5–7]. The presence of multiple *Se^390,418^*‐containing configurations further indicates that the Le(a+b+) phenotype cannot be attributed to a single *FUT2* diplotype. These findings may be particularly relevant to transfusion medicine in Thailand, as recognition of the population‐specific *FUT2–FUT3* genetic background may improve the interpretation of Le phenotypes and provide a more appropriate genetic context for Le blood group testing in Thai individuals.

The identification of the rare *Se(se)^676^* allele further expands the allelic heterogeneity underlying the Le(a+b+) phenotype. The c.676G>A (p.(Ala226Thr); rs1410377195) variant was extremely rare in gnomAD v4.1.1, with an allele frequency of 8.06 × 10^−6^ and no homozygous individuals reported [25]. The variant is located within the lumenal region of *α*(1,2)‐FucT2 and was not predicted by ProtVar to occur within a protein pocket or protein–protein interaction site [26]. However, the available computational evidence regarding its functional effect was inconsistent. Although Ala226 was highly conserved (conservation score, 0.96), CADD and ESM‐1b predicted a potentially deleterious effect, whereas FoldX predicted reduced protein stability. In contrast, AlphaMissense, popEVE, and Missense3D did not provide consistent evidence of functional impairment, and PolyPhen‐2 classified the variant as possibly damaging [27]. The predicted FoldX *ΔΔ*G of 2.44 kcal/mol suggests a potential reduction in protein stability; however, this computational prediction alone does not establish a functional consequence. Compared with the established weak‐secretor variant c.418A>T (p.(Ile140Phe); rs1047781) [35], which showed a higher PolyPhen‐2 score (0.978 vs. 0.785) and substantially greater predicted destabilization by FoldX (*ΔΔ*G, 13.54 vs. 2.44 kcal/mol), c.676G>A showed less consistent computational evidence for a functional effect. Therefore, although p.(Ala226Thr) may affect *α*(1,2)‐FucT2 activity or stability, its biological significance and contribution to the Le(a+b+) phenotype remain uncertain and require functional validation. Together with the other *FUT2* and *FUT3* variants identified in this study, these findings support a model in which the Le(a+b+) phenotype can arise from multiple *FUT2–FUT3* allele combinations rather than from a single predominant variant.

Several limitations should be considered when interpreting these findings. The sample size available for molecular analysis was relatively limited, particularly for rare *FUT2* and *FUT3* variants and specific genotype combinations, resulting in small numbers in some genetic subgroups and limiting definitive genotype–phenotype interpretation. A further limitation is that short‐read NGS cannot directly resolve complete allele sequences when multiple variants are present, which may limit definitive haplotype assignment. Definitive haplotype‐level characterization would require long‐range PCR combined with third‐generation, single‐molecule sequencing. In addition, the functional significance of the newly identified c.676G>A (p.(Ala226Thr)) variant remains uncertain in the absence of functional validation. RNA expression and enzymatic activity assays were not performed in the present study; therefore, the functional consequences of the identified variants could not be directly assessed, and the residual activity of weakened *FUT2* alleles could not be quantitatively determined. Functional studies will be required to definitively characterize the c.676G>A (p.(Ala226Thr)) variant and to further clarify the relationship between residual *α*(1,2)‐FucT2 activity and Le^b^ expression. Nevertheless, the relatively high frequency of the Le(a+b+) phenotype observed in the Thai population, together with the heterogeneous *FUT2–FUT3* genetic backgrounds identified in this study, indicates that population‐specific genetic variation should be considered when interpreting Le phenotypes. This may be particularly relevant in Thailand, where Le(a+b+) appears to occur more frequently than reported in several other populations, although differences in study populations, sample characteristics, and serological methods should be considered when making crosspopulation comparisons. Larger population‐based studies and functional characterization of rare variants will be required to validate these findings and further define their contribution to Le antigen expression. Future studies should also determine whether this population‐specific genetic information has practical utility in transfusion medicine, particularly for the interpretation of weak or discrepant Le serological phenotypes and for understanding the potential relationship between *FUT2–FUT3* genotype, Le antigen expression, and transfusion outcomes.

## 5. Conclusion

In conclusion, this study delineates the genetic architecture of *FUT2–FUT3* variation underlying Le phenotypes in the Thai population and provides a genetic explanation for the relatively high prevalence of Le(a+b+). In the Le(a+b+) cohort, all individuals carried the weakened *Se^390,418^* allele, predominantly in the homozygous state, whereas the remaining individuals carried *Se^390,418^* in combination with nonfunctional, deleted, or functionally uncertain *FUT2* alleles, demonstrating that multiple *FUT2* variant combinations can give rise to the Le(a+b+) phenotype. We also identified and molecularly characterized the large *FUT2* deletions, *se^del^* and *se^del2^*, providing the first report of these deletion alleles in the Thai population. These findings provide new insights into the genetic basis of Le antigen expression and the relatively high prevalence of Le(a+b+) in Thais and may contribute to a better understanding of Le phenotype diversity in transfusion medicine.

## Author Contributions

P.K.: data curation, resources, and writing—original draft. O.N.: supervision, project administration, and writing—review and editing. W.S.: data curation and resources. K.T.: resources. K.I.: funding acquisition, project administration, conceptualization, formal analysis, writing—original draft, and writing—review and editing.

## Funding

This study was supported by the Thammasat University Research Fund (TUFT 065/2569).

## Disclosure

All authors reviewed the results and approved the final version of the manuscript.

## Conflicts of Interest

The authors declare no conflicts of interest.

## Supporting Information

Additional supporting information can be found online in the Supporting Information section.

## Supporting information


**Supporting Information 1** Table S1: Nomenclature and corresponding Human Genome Variation Society (HGVS) and International Society of Blood Transfusion (ISBT) designations of *FUT2* and *FUT3* alleles observed in this study.

**Supporting Information 2** Table S2: Combined *FUT2* and *FUT3* diplotypes associated with Le phenotypes and agglutination strength categories in the Thai population (*N* = 151).

**Supporting Information 3** Figure S1: NGS read alignment showing *FUT2* deletion breakpoint junctions. Representative Integrative Genomics Viewer (IGV) plots showing the deletion breakpoint junctions of (a) *se^del^* in LEW062, corresponding to an approximately 10‐kb deletion; (b) *se^del2^* in LEW163, corresponding to an approximately 9.3‐kb deletion; and (c) *se^del^* in LEW234, corresponding to an approximately 10‐kb deletion.

**Supporting Information 4** Figure S2: Electropherograms of the *FUT2* gene showing the novel c.676G>A variant. DNA sequences are shown for (a) the c.676G/G genotype and (b) the c.676G/A genotype.

## Data Availability

The data that support the findings of this study are available on request from the corresponding author. The data are not publicly available due to privacy or ethical restrictions.
