# Supplementary material for: The Genetic Architecture of FUT2–FUT3 Variation and Variant Combinations Underlies the Relatively High Prevalence of the Le(a+b+) Phenotype in Thais
Source: Hum Mutat. 2026 Nov 10;2026:3749006. doi: 10.1155/humu/3749006 (PMC13751820; doi:10.1155/humu/3749006)
Supplement: Supplementary file 1 [file HUMU-2026-3749006-s001.docx]

**SUPPLEMENTARY DATA FIGURE S2:** Electropherograms of the *FUT2* gene showing the novel c.676G>A variant. DNA sequences are shown for a) the c.676G/G genotype and b) the c.676G/A genotype.Supporting Information
Additional supporting information can be found online in the Supporting
Information section. Supporting Information 1. The supporting ma-
terial file contains Table S1: (A) Clinical and treatment variable defi-
nitions and (B) genomic, follow-up, and outcome variable definitions;
Table S2: Representative targeted-panel content, quality-control thresh-
olds, software, and databases; and Table S3: Statistical analysis frame-
work. Supporting Information 2. Figure S1: Hypothesis-generating
genomic–microenvironment framework.
