# Supplementary material for: The Genetic Architecture of FUT2–FUT3 Variation and Variant Combinations Underlies the Relatively High Prevalence of the Le(a+b+) Phenotype in Thais
Source: Hum Mutat. 2026 Nov 10;2026:3749006. doi: 10.1155/humu/3749006 (PMC13751820; doi:10.1155/humu/3749006)
Supplement: Supplementary file 2 [file HUMU-2026-3749006-s002.docx]

**Supplemental Data Table S1 |** Nomenclature and corresponding Human Genome Variation Society (HGVS) and International Society of Blood Transfusion (ISBT) designations of *FUT2* and *FUT3* alleles observed in this study.

| **Gene** | **Functional class** | **HGVS variant description** | **Abbreviated nucleotide designation** | **ISBT allele designation** |
| --- | --- | --- | --- | --- |
| *FUT2* | Functional | Wild-type | *Se* | *FUT2*01* |
|  |  | c.390C>T | *Se^390^* | NA |
|  |  | c.[204A>G;249C>T] | *Se^204,249^* | NA |
|  |  | c.[351C>T; 390C>T] | *Se^351,390^* | NA |
|  |  | c.[390C>T;513C>T] | *Se^390,513^* | NA |
|  |  | c.[390C>T;749G>A] | *Se^390,749^* | NA |
|  |  | c.[204A>G;249C>T;642G>A] | *Se^204,249,642^* | NA |
|  | Reduced-function | c.418A>T | *Se^418^* | *FUT2*01W.02.01* |
|  |  | c.886G>A | *Se^886^* | *FUT2*01W.03* |
|  |  | c.[390C>T;418A>T] | *Se^390,418^* | NA |
|  |  | c.[311C>T;390C>T;418A>T] | *Se^311,390,418^* | NA |
|  |  | c.[390C>T;418A>T;567G>A] | *Se^390,418,567^* | NA |
|  |  | c.[390C>T;418A>T;642G>A] | *Se^390,418,642^* | NA |
|  |  | c.[390C>T;418A>T;749G>A] | *Se^390,418,749^* | NA |
|  | Loss-of-function | Large genomic deletion (~10 kb) | *se^del^* | *FUT2*0N.01* |
|  |  | c.232G>T | *se^232^* | NA |
|  |  | c.335C>T | *se^335^* | *FUT2*01N.15* |
|  |  | c.772G>A | *se^772^* | *FUT2*01N.16* |
|  |  | c.882G>A | *se^882^* | *FUT2*01N.12* |
|  |  | c.[390C>T;602G>A] | *se^390,602^* | NA |
|  |  | c.[390C>T;772G>A] | *se^390,772^* | NA |
|  |  | c.[204A>G;249C>T;335C>T] | *se^204,249,335^* | NA |
|  |  | c.[204A>G;249C>T;461G>A] | *se^204,249,461^* | NA |
|  |  | c.[204A>G;249C>T;772G>A] | *se^204,249,772^* | NA |
|  |  | c.[204A>G;249C>T;772G>A;993A>G] | *se^204,249,772,993^* | NA |
|  |  | c.[204A>G;249C>T;461G>A;772G>A;993A>G] | *se^204,249,461,772,993^* | NA |
| *FUT3* | Functional | Wild-type | *Le* | *FUT3*01.01* |
|  |  | c.1067T>A | *Le^1067^* | *FUT3*01.10* |
|  | Reduced-function | c.59T>G | *Le^59^* | *FUT3*01.17.01* |
|  | Loss-of-function | c.[59T>G;508G>A] | *le^59,508^* | *FUT3*01N.17.05* |
|  |  | c.[59T>G;1061G>A] | *le^59,1061^* | NA |
|  |  | c.[59T>G;1067T>A] | *le^59,1067^* | *FUT3*01N.17.03* |
|  |  | c.[202T>C;314C>T] | *le^202,314^* | *FUT3*01N.03.02* |
|  |  | c.[59T>G;508G>A;1061G>A] | *le^59,508,1061^* | *FUT3*01N.17.19* |
|  |  | c.[59T>G;508G>A;1067T>A] | *le^59,508,1067^* | NA |
|  |  | c.[202T>C;314C>T;1067T>A] | *le^202,314,1067^* | NA |

**Note:** *FUT2* and *FUT3* alleles were classified as functional, reduced-function, or loss-of-function according to the expected functional activity of the encoded α(1,2)-FucT2 and α(1,3/1,4)-FucT3, respectively.

**Abbreviations:** *Se* and *Le* denote functional alleles, whereas *se* and *le* denote nonfunctional alleles. HGVS, Human Genome Variation Society; ISBT, International Society of Blood Transfusion; NA, not assigned.
