# Supplementary material for: The Genetic Architecture of FUT2–FUT3 Variation and Variant Combinations Underlies the Relatively High Prevalence of the Le(a+b+) Phenotype in Thais
Source: Hum Mutat. 2026 Nov 10;2026:3749006. doi: 10.1155/humu/3749006 (PMC13751820; doi:10.1155/humu/3749006)
Supplement: Supplementary file 3 [file HUMU-2026-3749006-s003.docx]

**Supplemental Data Table S2 |** Combined *FUT2* and *FUT3* diplotypes associated with Le phenotypes and agglutination strength categories in the Thai population (N = 151).

| ***FUT2* diplotype** | | **Secretor status** | ***FUT3* diplotype** | | **Anti-Le^a^** | **Anti-Le^b^** | | **Le phenotype** | | **N** | **%** |
| --- | --- | --- | --- | --- | --- | --- | --- | --- | --- | --- | --- |
| *Se* | *Se* | Secretor | *Le* | *le^59,1067^* | 0 | 3+ | | Le(a−b+) | | 1 | 0.7 |
| *Se* | *Se^390^* |  | *Le* | *Le* | 0 | 3+ | |  | | 2 | 1.3 |
|  |  |  | *Le* | *Le^59^* | 0 | 3+ | |  | | 3 | 2.0 |
|  |  |  | *Le* | *le^59,508^* | 0 | 3+ | |  | | 1 | 0.7 |
|  |  |  | *Le* | *le^59,1067^* | 0 | 3+ | |  | | 3 | 2.0 |
|  |  |  | *Le^59^* | *le^59,1067^* | 0 | 3+ | |  | | 1 | 0.7 |
|  |  |  | *Le^59^* | *le^59,508,1067^* | 0 | 3+ | |  | | 1 | 0.7 |
| *Se* | *Se^390,418^* |  | *Le* | *Le* | 0 | 3+ | |  | | 1 | 0.7 |
|  |  |  | *Le* | *Le^59^* | 0 | 4+ | |  | | 2 | 1.3 |
|  |  |  | *Le* | *Le^1067^* | 0 | 3+ | |  | | 2 | 1.3 |
|  |  |  | *Le* | *le^59,1067^* | 0 | 4+ | |  | | 3 | 2.0 |
| *Se* | *Se^351,390^* |  | *Le* | *Le* | 0 | 3+ | |  | | 2 | 1.3 |
| *Se^390^* | *Se^390^* |  | *Le* | *Le^59^* | 0 | 3+ | |  | | 4 | 2.6 |
|  |  |  | *Le* | *Le^1067^* | 0 | 3+ | |  | | 4 | 2.6 |
|  |  |  | *Le* | *le^59,508^* | 0 | 4+ | |  | | 2 | 1.3 |
|  |  |  | *Le* | *le^59,1067^* | 0 | 3+ | |  | | 4 | 2.6 |
|  |  |  | *Le* | *le^202,314^* | 0 | 3+ | |  | | 1 | 0.7 |
|  |  |  | *Le^59^* | *le^59,1067^* | 0 | 3+ | |  | | 1 | 0.7 |
| *Se^390^* | *Se^204,249^* |  | *Le* | *Le^1067^* | 0 | 3+ | |  | | 1 | 0.7 |
| *Se^390^* | *Se^390,418^* |  | *Le* | *Le* | 0 | 4+ | |  | | 7 | 4.6 |
|  |  |  | *Le* | *Le^59^* | 0 | 2+ | |  | | 11 | 7.3 |
|  |  |  | *Le* | *Le^1067^* | 0 | 3+ | |  | | 2 | 1.3 |
|  |  |  | *Le* | *le^59,508^* | 0 | 3+ | |  | | 10 | 6.6 |
|  |  |  | *Le* | *le^202,314^* | 0 | 3+ | |  | | 1 | 0.7 |
|  |  |  | *Le* | *le^59,1061^* | 0 | 3+ | |  | | 1 | 0.7 |
|  |  |  | *Le* | *le^59,1067^* | 0 | 4+ | |  | | 4 | 2.6 |
|  |  |  | *Le^59^* | *le^59,508,1067^* | 0 | 3+ | |  | | 1 | 0.7 |
|  |  |  | *Le^1067^* | *le^59,1067^* | 0 | 3+ | |  | | 1 | 0.7 |
| *Se^390,418^* | *Se^390,513^* |  | *Le* | *Le* | 0 | 3+ | |  | | 1 | 0.7 |
|  |  |  | *Le* | *Le^1067^* | 0 | 3+ | |  | | 1 | 0.7 |
| *Se^390,749^* | *Se^390,418,749^* |  | *Le* | *Le* | 0 | 3+ | |  | | 1 | 0.7 |
| *Se^390^* | *Se^204,249,642^* |  | *Le* | *Le^59^* | 0 | 3+ | |  | | 1 | 0.7 |
| *Se^390^* | *Se^311,390,418^* |  | *Le* | *le^59,508^* | 0 | 3+ | |  | | 1 | 0.7 |
| *Se^390^* | *Se^390,513^* |  | *Le* | *le^59,508^* | 0 | 3+ | |  | | 1 | 0.7 |
|  |  |  | *Le* | *le^202,314^* | 0 | 3+ | |  | | 1 | 0.7 |
| *Se* | *se^204,249,772,993^* |  | *Le* | *Le* | 0 | 3+ | |  | | 1 | 0.7 |
| *Se^390^* | *se^204,249,772,993^* |  | *Le* | *le^59,508^* | 0 | 3+ | |  | | 1 | 0.7 |
|  |  |  | *Le* | *le^202,314^* | 0 | 3+ | |  | | 1 | 0.7 |
| **Total Le(a−b+)** | | | | | | | | | | **87** | **57.6** |
| *Se^390,418^* | *Se^390,418^* | Weak-secretor | *Le* | *Le* | 3+ | | 2+ | | Le(a+b+) | 10 | 6.6 |
|  |  |  | *Le* | *Le^59^* | 3+ | | 2+ | |  | 3 | 2.0 |
|  |  |  | *Le* | *Le^1067^* | 2+ | | 2+ | |  | 5 | 3.3 |
|  |  |  | *Le* | *le^59,508^* | 3+ | | 2+ | |  | 2 | 1.3 |
|  |  |  | *Le* | *le^59,1067^* | 2+ | | 2+ | |  | 1 | 0.7 |
|  |  |  | *Le* | *le^59,508,1061^* | 3+ | | 1+ | |  | 1 | 0.7 |
|  |  |  | *Le* | *le^59,508,1067^* | 3+ | | 2+ | |  | 1 | 0.7 |
|  |  |  | *Le^59^* | *le^202,314^* | 3+ | | 3+ | |  | 1 | 0.7 |
| *Se^418^* | *Se^390,418^* |  | *Le* | *Le* | 3+ | | 3+ | |  | 1 | 0.7 |
|  |  |  | *Le* | *Le^1067^* | 3+ | | 2+ | |  | 1 | 0.7 |
| *Se^390,418^* | *Se^390,418,567^* |  | *Le* | *le^202,314^* | 2+ | | 2+ | |  | 1 | 0.7 |
| *Se^390,418^* | *Se^886^* |  | *Le* | *Le* | 3+ | | 2+ | |  | 1 | 0.7 |
| *Se^390,418,642^* | *Se^390,418,642^* |  | *Le* | *Le* | 2+ | | 2+ | |  | 1 | 0.7 |
|  |  |  | *Le* | *le^59,1067^* | 3+ | | 2+ | |  | 1 | 0.7 |
| *Se^390,418^* | *se^del^* |  | *Le* | *Le* | 3+ | | 2+ | |  | 1 | 0.7 |
| *Se^390,418^* | *se^204,249,772^* |  | *Le* | *Le* | 3+ | | 1+ | |  | 1 | 0.7 |
|  |  |  | *Le* | *Le^59^* | 3+ | | 1+ | |  | 1 | 0.7 |
| *Se^390,418^* | *se^232^* |  | *Le* | *le^202,314^* | 2+ | | 1+ | |  | 1 | 0.7 |
| *Se^390,418^* | *se^390,602^* |  | *Le* | *Le* | 2+ | | 2+ | |  | 1 | 0.7 |
| *Se^390,418^* | *se^882^* |  | *Le* | *Le* | 3+ | | 2+ | |  | 1 | 0.7 |
| **Total Le(a+b+)** | | | | | | | | | | **36** | **23.8** |
| *Se^390^* | *Se^390^* | Secretor | *le^59,1067^* | *le^59,1067^* | 0 | | 0 | | Le(a−b−) | 2 | 1.3 |
|  |  |  | *le^59,508,1067^* | *le^59,508,1067^* | 0 | | 0 | |  | 1 | 0.7 |
| *Se* | *Se^390,418^* |  | *le^59,1067^* | *le^59,1067^* | 0 | | 0 | |  | 2 | 1.3 |
| *Se^390^* | *Se^390,418^* |  | *le^59,1067^* | *le^59,1067^* | 0 | | 0 | |  | 5 | 3.3 |
|  |  |  | *le^59,1067^* | *le^202,314,1067^* | 0 | | 0 | |  | 1 | 0.7 |
|  |  |  | *le^59,1067^* | *le^59,508,1061^* | 0 | | 0 | |  | 1 | 0.7 |
|  |  |  | *le^59,1067^* | *le^59,508,1067^* | 0 | | 0 | |  | 1 | 0.7 |
| *Se^390,418^* | *Se^390,418^* | Weak-secretor | *le^202,314^* | *le^202,314^* | 0 | | 0 | |  | 1 | 0.7 |
|  |  |  | *le^59,1067^* | *le^59,1067^* | 0 | | 0 | |  | 4 | 2.6 |
|  |  |  | *le^59,508,1067^* | *le^59,508,1067^* | 0 | | 0 | |  | 1 | 0.7 |
|  |  |  | *le^59,508^* | *le^59,1067^* | 0 | | 0 | |  | 1 | 0.7 |
| *Se^418^* | *Se^390,418^* |  | *le^59,1067^* | *le^59,1067^* | 0 | | 0 | |  | 1 | 0.7 |
| *Se^390,418^* | *se^335^* |  | *le^59,1067^* | *le^59,1067^* | 0 | | 0 | |  | 1 | 0.7 |
| *se^390,772^* | *se^204,249,772^* | Non-secretor | *le^59,508^* | *le^59,1067^* | 0 | | 0 | |  | 1 | 0.7 |
| *se^204,249,461,772,993^* | *se^204,249,461,772,993^* |  | *le^59,1067^* | *le^59,1067^* | 0 | | 0 | |  | 1 | 0.7 |
| **Total Le(a−b−)** | | | | | | | | | | **24** | **15.9** |
| *se^335^* | *se^335^* | Non-secretor | *Le* | *Le* | 3+ | | 0 | | Le(a+b−) | 1 | 0.7 |
| *se^204,249,335^* | *se^772^* |  | *Le* | *le^59,508,1067^* | 2+ | | 0 | |  | 1 | 0.7 |
| *se^390,418,461^* | *se^204,249,772,993^* |  | *Le* | *le^59,508,1067^* | 2+ | | 0 | |  | 1 | 0.7 |
| *se^772^* | *se^204,249,772^* |  | *Le* | *Le* | 2+ | | 0 | |  | 1 | 0.7 |
| **Total Le(a+b−)** | | | | | | | | | | **4** | **2.6** |
