# Supplementary material for: The Genetic Architecture of FUT2–FUT3 Variation and Variant Combinations Underlies the Relatively High Prevalence of the Le(a+b+) Phenotype in Thais
Source: Hum Mutat. 2026 Nov 10;2026:3749006. doi: 10.1155/humu/3749006 (PMC13751820; doi:10.1155/humu/3749006)
Supplement: Supplementary file 4 [file HUMU-2026-3749006-s004.docx]

**SUPPLEMENTARY DATA FIGURE S1:** NGS read alignment showing *FUT2* deletion breakpoint junctions. Representative Integrative Genomics Viewer (IGV) plots showing the deletion breakpoint junctions of a) *se^del^* in LEW062, corresponding to an approximately 10-kb deletion; b) *se^del2^* in LEW163, corresponding to an approximately 9.3-kb deletion; and c) *se^del^* in LEW234, corresponding to an approximately 10-kb deletion. The 5′ breakpoint (5′ BP) and 3′ breakpoint (3′ BP) indicate the deletion boundaries, and dashed lines indicate the deleted genomic regions. Supporting Information
Additional supporting information can be found online in the Supporting
Information section. Supporting Information 1. The supporting ma-
terial file contains Table S1: (A) Clinical and treatment variable defi-
nitions and (B) genomic, follow-up, and outcome variable definitions;
Table S2: Representative targeted-panel content, quality-control thresh-
olds, software, and databases; and Table S3: Statistical analysis frame-
work. Supporting Information 2. Figure S1: Hypothesis-generating
genomic–microenvironment framework.
